# Difficult Cases

**Published:** 1886-10-09

**Authors:** 


### Difficult Cases.

*The intention of this column is to afford assistance to clergymen, district visitors, hospital officials, medical men, and others who have to provide for the treatment or accommodation of difficult cases of disease, sometimes in its acute, but usually in its chronic form. Anyone being in doubt as to the best course to pursue in any such case, is invited to communicate with the Editor. All such communications should be written on one side of the paper only, and be addressed to the Editor, the envelope being marked in the top left-hand corner, "Difficult Cases."*

### An Elderly Blind Gentleman.

Dr. A. writes: "Could you tell me of a home within easy reach of London, where an elderly gentleman, who has lost his eyesight, could be received at a moderate rate?"

*** Dr. A. should write to Dr. T. R. Armytage, Hon. Sec. British and Foreign Blind Association, 33, Cambridge Square, Hyde Park, W.

THE ADMISSION OF PATIENTS SUFFERING FROM FITS TO ASYLUMS FOR IDIOTS.

J. F. W. asks if patients subject to fits are eligible for admission to idiot asylums.

*** Epileptic idiots and imbeciles, whose friends are able to pay remunerative rates for their maintenance, are sometimes received at Earlswood and other idiot asylums, but an enhanced rate of payment is usually required, owing to the extra care and attention which are necessary when epilepsy exists as a complication. For epileptic idiot children of the poorer classes no suitable accommodation exists, as that provided in workhouses and public lunatic asylums is not adapted to the requirements of such patients. Those occurring in the metropolitan area constitute an exception to the latter statement, because the metropolitan district schools at Darenth admit idiotic and imbecile children, whether epileptic or not, upon the order of the poor-law guardians of the Metropolitan Unions.

DEFECTIVE MENTAL CAPACITY.

M.D. writes: "I shall be grateful if any of my professional brethren will advise me what is best to do (educationally) with a youth, aged thirteen, not idiotic, but yet of such defective mental capacity that it would be out of the question to send him to any ordinary school. He is not more difficult to control than such cases usually are. He is the only child of a widow with very limited means."

*** There is no institution exactly suitable for such a case as this. Inquiry might be made to the Medical Superintendent, Royal Albert Asylum, Lancaster, or to the Midland Counties Idiot Asylum, Knowles, Birmingham; or at one of the other county asylums, at Colchester, Earlswood, or Exeter, according to the district in which the child's parent resides.

